# A new epidemic wave of *Bordetella pertussis* in paediatric population: impact and role of co-infections in pertussis disease

**DOI:** 10.1186/s13052-025-01865-4

**Published:** 2025-01-21

**Authors:** Rossana Scutari, Giulia Linardos, Stefania Ranno, Mara Pisani, Anna Chiara Vittucci, Luana Coltella, Luna Colagrossi, Velia Chiara Di Maio, Annamaria Sisto, Livia Mancinelli, Simona Landi, Sebastian Cristaldi, Massimiliano Raponi, Paola Bernaschi, Alberto Villani, Cristina Russo, Carlo Federico Perno

**Affiliations:** 1 Unit of Microbiology and Diagnostic Immunology, Bambino Gesù Children’s Hospital, IRCSS, Rome, 00165 Italy; 2 Multimodal Laboratory Research Unit, Bambino Gesù Children’s Hospital, IRCCS, Rome, 00165 Italy; 3 Hospital University Pediatrics Clinical Area, Bambino Gesù Children’s Hospital IRCCS, Rome, Italy; 4 Medical Direction, Bambino Gesù Children’s Hospital, IRCCS, Rome, 00165 Italy

**Keywords:** *Bordetella pertussis*, Whooping cough, Co-infections, Infants, Epidemiology, Pertussis disease

## Abstract

**Background:**

In recent months, *Bordetella pertussis* has reappeared after maintaining a low rate for many years. Although pertussis is usually characterized by a favorable course, several factors can contribute to the severity of the disease, such as mixed respiratory infections. In this study, we evaluate *B.pertussis* cases observed in the pediatric population followed at the Bambino Gesù Children's Hospital and analyzed the potential impact of co-infections in relation to disease severity.

**Methods:**

From January to May 2024, a total of 1,151 children and adolescents (both inpatients and outpatients) were screened for the presence of respiratory pathogens, including *B.pertussis,* with clinically relevant respiratory symptoms.

**Results:**

Among the 1,151 patients screened, 66 tested positive for *B.pertussis*. Fourteen patients had respiratory failure, and six of them required intensive care unit (ICU) admission, while 52 had mild infection. 23.3% of patients had *B.pertussis* alone, while 76.7% had co-infections (including 5 patients admitted to the ICU). A higher co-infection rate was observed in patients with respiratory failure than in those without failure (92.9% vs. 69.0%, *p*-value:0.041). Rhinovirus, Metapneumovirus and Parainfluenza-virus were the most prevalent in our pediatric population. Co-infections of human bocavirus with *B.pertussis* were observed exclusively in patients with respiratory failure.

**Conclusions:**

Our results highlighted an increase in *B.pertussis* cases from January to May 2024, reaching a peak of cases in the month of May. This study shows a high rate of *B.pertussis* co-infection, and a trend toward association between *B.pertussis* and specific viruses, that might play a role in increasing disease severity.

**Supplementary Information:**

The online version contains supplementary material available at 10.1186/s13052-025-01865-4.

## Background

*Bordetella pertussis* (*B.pertussis*) is a Gram-negative, pleomorphic, aerobic coccobacillus [1]. This bacterium produces a number of virulence factors, including pertussis toxin, adenylate cyclase toxin, filamentous hemagglutinin, and hemolysin [2]. *B.pertussis* is the causative pathogen of pertussis or whooping cough, a highly contagious respiratory disease characterized by paroxysmal coughing that ends in prolonged wheezing and may be associated with complications (even fatal) [3]. As the clinical presentation can be similar to that of other respiratory diseases, accurate diagnosis of pertussis requires laboratory confirmation, usually by molecular methods. Once pertussis is diagnosed, other potential causative pathogens are rarely examined; however, this does not exclude their coexistence [4]. Nowadays, the role of mixed *B.pertussis* co-infections in the pathogenesis of disease is not clear [5].

Pertussis is a preventable disease with the administration of an acellular vaccine composed of purified components of the organism *B.pertussis* and detoxified pertussis toxin. The infection affects individuals of all ages; while infants are at risk for severe pertussis, children, adolescents, and adults, even if vaccinated, can be infected with *B.pertussis* and are known to be the main reservoirs of the bacterium [6]. The change in the mode of transmission, from the previous child-child epidemic mode to an adult–child epidemic mode after the introduction of the immunization program, makes infants and young children more susceptible to infection, particularly of not-vaccinated [7]. For this reason, it is critical that family members of children who cannot receive the vaccine be immunized. Of note, administration of Tetanus, Diphtheria, Pertussis Vaccine (Tdap) between 27 and 36 weeks of pregnancy reduces the risk of pertussis in babies younger than 2 months old by 78%, and is associated with a reduction in serious complications including hospitalization (https://www.cdc.gov/pertussis/pregnant/mom/get-vaccinated.html). These immunization strategies have led to a decrease in the number of pertussis cases, however, even in countries with broad vaccination coverage, such as Italy, the incidence of pertussis has returned to an upward trend after maintaining a low rate for many years. For this reason, whooping cough continues to pose a threat to public health. This was recently demonstrated with a fatal case of pertussis in a neonate with pulmonary hypertension and respiratory failure [8]. Here we describe and evaluate the *B.pertussis* outbreak observed in the paediatric population followed at the Bambino Gesù Children's Hospital from January to May 2024, with the aim of studying and analysing *B.pertussis* co-infection and its relationship with clinical severity.

## Methods

### Study design and setting

This retrospective observational study includes 1,242 respiratory samples from 1,151 children and adolescents (0–18 years, both inpatients and outpatients) referred to Bambino Gesù Children's Hospital, Rome, Italy, for the presence of clinically relevant respiratory symptoms from January, 2024 to May, 2024. Of these, 66 tested positive for *B.pertussis.*

The patients included in the study were from the emergency department (emergency room), outpatients and hospital wards. In our pediatric hospital, the criteria for admission to the ward or intensive care unit are: young age, respiratory distress, vomiting, cyanosis, apnea, feeding difficulties, lethargy, poor family compliance, increased white blood cells [9].

Nasopharyngeal swabs and nasopharyngeal aspirates were collected from each patient and analysed at the Microbiology Unit according to standard laboratory operating procedures.

### Laboratory testing

*B.pertussis* identification was performed by both a syndromic approach FilmArray (BioFire® Respiratory 2.1 plus [RP2.1plus] Panel, Biomerieux, Marcy-l’Étoile, France) and single target real-time PCR for *B.pertussis* (Bordetella R-gene®, Biomerieux, Marcy-l’Étoile, France) according to the manufacturer’s recommendation.

The BioFire® Respiratory 2.1 plus Panel detect *B.pertussis* with a limit of detection of 1.0 × 10^3^ CFU/mL. Differently, Bordetella R-gene has as limit of detection (LoD 95%) of 250 bacteria/mL.

Both methods, in addition to detection of the bacterium, allow estimation of the bacterial load expressed as Cycle threshold (Ct) in the single target real-time PCR or crossing points (Cp) in the BioFire® system.

For nasopharyngeal aspirate samples, culture was also performed, on specialized Bordetella-selective agar (Biolife, Milan, Italy) and Bordet Gengou (BD) and plates were incubated under aerobic conditions at 37 °C. Suspected *B.pertussis* colonies were confirmed using MALDI-TOF MS (Bruker, Billerica, MA, USA).

*B.pertussis* results were supplemented with Allplex Respiratory Panel Assay or FilmArray to identify viral co-infection and with standard growth media for culturing respiratory microorganisms for bacterial co-infection.

### Statistical analysis

Descriptive statistics are expressed as median values and interquartile range (IQR) for continuous data and number (percentage) for categorical data. Statistical comparisons were performed using Fisher’s exact and Mann–Whitney tests for categorical and continuous variables, respectively. A value of p less than 0.05 was considered statistically significant.

Data were analysed using statistical software package SPSS (v32.0; SPSS Inc., Chicago, IL, USA).

## Results

### Epidemiology

From January, 01 to May, 31 2024, a total of 1,242 respiratory samples from 1,151 children and adolescents were screened for respiratory pathogens including *B.pertussis* at the Bambino Gesù Children's Hospital in Rome, Italy. During this period, we observed a marked increase in the detections of *B.pertussis,* the maximum frequency being recorded in May. Specifically, a diagnosis of *B.pertussis*, confirmed by positive molecular assays, was made in 66 patients (Fig. S1); 39.4% (*N* = 26) of patients were from the outpatient department and thus had suspected pertussis, while 51.5% (*N* = 34) were from the emergency department with respiratory symptoms of varying intensity (cough, apnea or dyspnea). The remaining 9.1% (*N* = 6) of patients came from the hospital wards.

### General population

The majority of patients (*N* = 37, 56.1%) at diagnosis had less than one year (28.8% had < 3 months, 18.2% had 3- < 6 months, and 9.1% had 6–12 months), while 29 (43.9%) had > 1 year.

More than half of the patients (*N* = 36, 54.5%) were hospitalized for a median (IQR) duration of hospitalization of 8 (4–10) days. Of these, 6 (9.1%) required admission to the intensive care unit (ICU). Overall, 44 (66.8%) paediatric patients did not require ventilatory support, while the remaining 22 needed respiratory support. Specifically, 8 (12.1%) patients required low flow oxygen, 9 (13.6%) needed high flow nasal cannula, 2 (3.0%) and 3 (4.5%) patients required Helmet continuous positive airway pressure and mechanical ventilation, respectively. Regarding vaccination status, which was available for 45 patients, 23 (51.1%) patients were too young to be vaccinated, while only 17 (37.8%) had received total or partial number of pertussis vaccine doses; 5 (11.1%) patients had not been vaccinated by parental decision. For 21 patients, vaccination status was unknown. Only 4 of the patients’ mothers had received Tdap vaccine before or during the current pregnancy.

Demographic and clinical characteristics are reported in Table 1.

**Table 1:** Demographic and clinical characteristics of patients according to respiratory failure

	Overall	Respiratory failure	Non-respiratory failure	*P*-value
N	66	14	52	
Male, N (%)	33 (50.0)	10 (71.4)	23 (44.2)	0.134
Age, median (IQR)	0.59 (0.21–5.12)	0.21 (0.12–0.41)	2.2 (0.28–5.55)	**< 0.001**
Age, N (%)				
< 3 months	19 (28.8)	9 (64.3)	10 (19.2)	**0.002**
3- < 6 months	12 (18.2)	2 (14.3)	10 (19.2)	1.000
6 months—< 12 months	6 (9.1)	2 (14.2)	4 (4.0)	0.600
> 12 months	29 (43.9)	1 (7.0)	28 (53.8)	**0.002**
*B.pertussis* load (Ct/Cp), median (IQR)	25.8 (20.7–30.9)	28.0 (20.4–32.5)	25.8 (20.9–30.0)	0.535
Vaccination, total/partial, N (%) (*N* = 45)	17 (37.8)	5 (35.7)	12 (38.7)	1.000
Vaccine during pregnancy, N (%) (*N* = 32)	4 (12.5)	1 (7.7)	3 (15.8)	0.629
***Clinical features***				
No respiratory support needed, N (%)	44 (66.8)	0 (0.0)	44 (84.6)	**< 0.001**
Oxygen low flow, N (%) (*N* = 61)	8 (12.1)	0 (0.0)	8 (17.0)	0.187
High Flow Nasal Cannula, HFNC, N (%)	9 (13.6)	9 (64.3)	-	-
Helmet continuous positive airway pressure, hCPAP, N (%)	2 (3.0)	2 (14.3)	-	-
Mechanical ventilation, VM, N (%)	3 (4.5)	3 (21.4)	-	-
White blood cells count, cells/mm^3^, median (IQR) (*N* = 37)	16,980 (13,730–24830)	21,760 (15,288–30,340)	16,190 (9970–15630)	0.062
Leukocytosis, n (%) (*N* = 37)	28 (75.7)	13 (92.9)	15 (65.2)	0.063
Hospitalization, N (%)	36 (54.5)	14 (100)	22 (42.3)	**< 0.001**
Intensive care, N (%)	6 (9.1)	6 (42.9)	-	-

*IQR* interquartile range; * two-sided *p*-values were calculated by Fisher’s exact test or Mann–Whitney test, as appropriate

### Population stratified according to respiratory failure

Looking at the severity of infection, 14 (21.2%) patients had respiratory failure, while 52 (78.8%) patients had a mild infection without respiratory failure.

By comparing the demographic and clinical characteristics in these two groups, we observed that patients with respiratory failure were significantly younger than those without respiratory failure (median [IQR] age: 0.21 [0.12–0.41] *vs* 2.2 [0.28–5.55] years, *p*-value < 0.001). In contrast, patients aged > 12 months belonged more to the group of patients without respiratory failure (53.8% no respiratory failure vs. 7.0% respiratory failure, *p*-value: 0.002).

As expected, patients with respiratory failure were all hospitalized (*N* = 14, 100%) while only 22 (42.3%) patients without respiratory failure required hospitalization (Table 1). Of note, the majority of the latter patients had less than 1-year-old (*N* = 18/22).

Regarding *B.pertussis* load, similar PCR Ct/Cp values were observed between two groups (median [IQR] 28.0 [20.4–32.5] Ct/Cp in respiratory failure compared to 25.8 [20.9–30.0] Ct/Cp in non-respiratory failure, *p*-value: 0.535) (Table 1).

Nine (64.3%) patients with respiratory failure required High Flow Nasal Cannula (HFNC), and 2 (14.3%) patients required Helmet continuous positive airway pressure (hCPAP). Mechanical ventilation was required in 3 (21.4%) patients.

In addition, patients with respiratory failure had a higher white blood cell count and a higher rate of leukocytosis than those without respiratory failure (white blood cell count, median [IQR]: 21760 [15288–30340] vs. 16190 [9970–15630] cells/mm^3^, *p*-value 0,064; leucocytosis, n [%]: 13 [92.9%] vs. 15 [65.2%], *p*-value: 0.062).

### Co-infection in general population

**Fig. 1:**
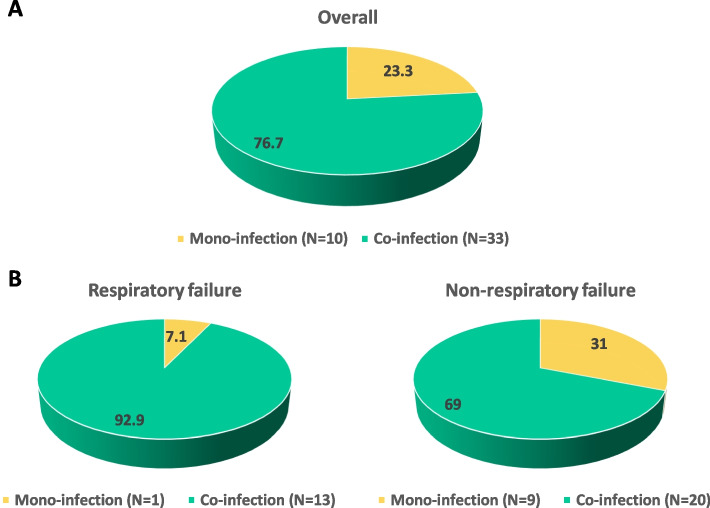
Distribution of microorganism detections in overall population (panel **A**) and according to respiratory failure (panel **B**)

Of the 66 patients with pertussis infection enrolled, 43 had concomitantly required evaluation of other respiratory microorganism. Of these, 10 (23.3%) patients (median [IQR] age of 0.29 [0.09–2.11] years) had *B.pertussis* alone, while 33 (76.7%) patients (median [IQR] age: 0.28 [0.20–0.87] years, *p* value: 0.857) were characterized by respiratory co-infections (Fig. 1, panel A).

By characterizing the type of co-infection, the majority involved viruses (*N* = 30, 90.9%), while bacterial co-infections (*Haemophilus influenzae* + *Staphylococcus aureus* and *Mycoplasma pneumoniae*) were observed in 2 (6.1%) patients. Only one patient, in addition to *B.pertussis*, had a co-infection characterized by both bacteria and virus (*Haemophilus influenzae* + *Moraxella catarrhalis* + Human Rhinovirus/Enterovirus).

Among the 33 patients with co-infection, 19 had co-infection with only one other pathogen (Human Rhinovirus/Enterovirus [*N* = 7], Human Rhinovirus [*N* = 5], Metapneumovirus [*N* = 3], Parainfluenza virus type 3 (PIV3) [*N* = 1], Coronavirus OC43 [*N* = 1], Respiratory Syncytial Virus [*N* = 1] and *Mycoplasma pneumoniae* [*N* = 1]), while 14 patients had multiple co-infections. Of interest, no demographic and clinical differences were observed between *B.pertussis* mono- and co-infections. A trend toward a lower Ct/Cp values was observed in mono-infection with respect to co-infection (median [IQR]: 20.7 [18.0–25.0] *vs* 25.4 [19.1–30.5] Ct/Cp, *p*-value:0.071). Similarly, no difference in term of co-infection was found between vaccinated- (or their mothers) and unvaccinated children.

### Co-infection in population stratified according to respiratory failure

Looking at the distribution of mono- and co-infections according to respiratory failure, a higher prevalence of co-infections was found in patients with respiratory failure than in those without respiratory failure (92.9% vs. 69.0%, *p*-value: 0.041) (Fig. 1, panel B). In both groups, co-infection with Rhinovirus alone or associated with other pathogens was most prevalent (6/13 [46.1%] in respiratory failure and 9/20 [45.0%] in non-respiratory failure) followed by Parainfluenza virus type 3 (3/13 [23.1%] in respiratory failure and 3/20 [15.0%] in non-respiratory failure). Of note, Human bocavirus co-infections were observed exclusively in patients with respiratory failure (3/13, 23.1%).

### Patients admitted to ICU

Concerning the 6 patients who required intensive care admission, 5/6 patients were less than 3 months old and thus had not received the vaccine; also their pregnant mothers had not received the vaccine. The last patient, 10 months old and with a diabetic ketoacidosis, had received 2 doses of vaccine. Overall, only one of these patients had presented to the hospital with suspected pertussis.

Of note, the bacterial load was higher in these patients than in other patients with respiratory failure who did not need ICU admission (Cp/Ct: 21.6 [15.6–28.0] *vs.* 30.8 [26.6–35.3], *p*-value:0.061).

Of interest, among these paediatric patients requiring admission to ICU, 5 patients were characterized by co-infections (*Haemophilus influenzae* + *Staphylococcus aureus,* Metapneumovirus, Human Rhinovirus + Respiratory Syncytial Virus, Human Rhinovirus/Enterovirus and Adenovirus + Human Rhinovirus/Enterovirus).

## Discussion

Pertussis remains a public health problem worldwide, even in countries where immunization coverage is high (https://www.who.int/teams/health-product-policy-and-standards/standards-and-specifications/vaccine-standardization/pertussis). In recent months, increasing cases of pertussis have also been reported in adolescents and adults (https://www.ecdc.europa.eu/en/publications-data/increase-pertussis-cases-eueea), who represent a reservoir for *B.pertussis* and the source of infection for susceptible infants and children for whom pertussis can be a life-threatening disease. In line with global observations, in this study we observed an increase in *B.pertussis* positivity cases from January to May 2024, reaching a peak of cases in the month of May. The increase in this infection and disease severity may be associated with several factors, including genetic changes in circulating *B.pertussis* strains, reduced vaccination coverage, and evidence that neither natural infection nor vaccination confers lifelong immunity. Several studies have looked at factors associated with disease severity, including age, bacterial load, vaccination status, and white blood cell count [10]. Despite this, the impact of the presence of possible co-infections has not been thoroughly evaluated to date.

In this study, we observed that 21.2% of children with pertussis diagnosed at our hospital had respiratory failure, most of them being < 3 months of age. It is well known that infants younger than 3 months of age are vulnerable to pertussis infection because they are too young to be vaccinated and may be associated with more serious complications (such as length of hospital stay and ICU admission). In line with this, we observed that 5 of the 6 patients who required access to intensive care were not vaccinated due to their very young age. It is known that an incompletely competent immune system characterising these patients can lead to an inadequate response to infections or pathogenic stimuli, with the risk of developing new infections and more severe clinical manifestations. This finding further highlights the important role of vaccine-induced immunity in the progression and severity of *pertussis* disease. Therefore, it appears necessary to encourage vaccination in pregnant women and family members closest to the newborn. A recent study showed that 64% maternal vaccination coverage led to a 78% reduction in pertussis cases and a 68% reduction in hospital admissions for children under 3 months [11]. Not to be underestimated is the fact that the severity of complications has an inevitable impact on the management of the hospitalized patient in terms of costs associated with hospitalization and care.

Regarding bacterial load, in our study no significant differences were shown in patients with respiratory failure and those without. Of note, some studies observed strong positive associations between *B.pertussis* nasopharyngeal bacterial load and clinical severity, as well as occurrence of complications [12–14]. This difference with our data could be related to the time of the quantitative evaluation of bacterial load. Indeed, in the course of *B.pertussis* infection*,* symptoms occur after the peak presence of bacteria, therefore the time of performing the test makes the difference in term of quantitative evaluation. In addition, nearly all of our patients have already started empiric antibiotic therapy before the molecular test, so the bacterial load at the time of molecular test might have been affected by this treatment. In line with this observation, cultures performed to detect *B.pertussis* were positive only in nine patients. Furthermore, focusing on the 6 most severe patients who needed ICU access, we observed higher *B.pertussis* DNA levels. Therefore, it is conceivable that indeed the bacterial load is associated with the severity of the infection, provided that the molecular test is performed early during the course of the infection. Further studies, including analysis of the effects of antibiotics on bacterial load, are needed to determine the prognostic value of *B.pertussis* DNA load in disease severity. The present study demonstrates a high prevalence of *B.pertussis* respiratory co-infections (76.7%) involving mostly younger patients, compared with infections characterized by *B.pertussis* alone. Slightly lower prevalence of *B.pertussis* co-infection (47%) was previously observed in infants hospitalized with pertussis over two years of observation [5]. In agreement with previous studies [5, 15], in our population the detection of *B.pertussis* in combination with other pathogens was most associated with viruses (90.9%). The coinfection prevalence with *B.pertussis* in this study (76.7%) is higher than the rate of general coinfections found in a pediatric population affected by respiratory disease, recently studied by our group, and performed during the same time period, (about 40%) [16]. The reasons for this high rate of co-infection requires further investigation; one hypothesis is that this bacterium, in combination with an incomplete immune system, might create a favourable environment for other pathogens infection, thus generating conditions for a more severe clinical outcome.

Of note, a higher co-infection rate was observed in patients with respiratory failure than those without (92.9% vs. 69.0%). If these results be confirmed in a large cohort of patients, it would advocate for an extensive use of multiplex test to identify the patients that, being coinfected, might be at higher risk of respiratory failure.

Virological analysis showed that only a few respiratory microorganisms are specifically associated ith co-detection of *B.pertussis*. Although most of the potential respiratory pathogens studied did not show a statistically significant association with the presence of *B.pertussis*, three, namely Human Rhinoviruses, Metapneumoviruses, and Parainfluenza virus type 3, were found to be most associated with *B.pertussis* co-detection in respiratory and non-respiratory failure settings. The distribution of co-infections could reflect the observation that the epidemiology of HRV maintains a constant epidemic curve throughout the year. Regarding Parainfluenza viruses, Muloiwa et al., observed that patients with *B.pertussis* had twice the risk of detecting parainfluenza viruses [4].

The role of co-infections in clinical practice remains debated. Whereas some studies suggest that viral co-infections with *B.pertussis* do not change the severity of the clinical picture [17–19], on the other hand, mixed respiratory tract infections may be responsible for more severe illness. In fact, viral or bacterial coinfection has been shown to be associated with severity of illness and length of hospitalization in children hospitalized for whooping cough [20, 21].

Our study has some limitations that need to be discussed. First, the monocentric nature of the study prevented us to collect a large number of clinical samples, limiting the possibility to draw certain statements. This limitation makes the results of the study exploratory. Therefore, a multicentre study with a larger sample size could be helpful and supportive to confirm the results obtained.

## Conclusions

The exploratory results of the present study, involving a small number of paediatric patients, showed a high rate of viral co-infection and a tendency for associations between B. pertussis and specific viruses in the overall population, particularly in patients with greater severity of disease associated with respiratory failure.

In light of the above, given the potential contribution of viral co-infections on the clinical severity of pertussis in infants, the search for other pathogens in a patient diagnosed with pertussis could have an important diagnostic and prognostic role. Therefore, in-depth microbiological analysis, and studying the dynamics and type of co-infection and their role in the severity of pertussis disease, might be critical for understanding the circulation of specific microorganisms, the impact of preventive immunization strategies, and, most importantly, for clinical decisions that enable early and specific treatment to prevent any serious complications.

## Supplementary Information

Supplementary Material 1: Supplementary figure 1. Temporal distribution of *B.pertussis* in the pediatric population from January 01, 2024 to May 31, 2024 admitted to the Bambino Gesù Children's Hospital in Rome, Italy.

## Data Availability

The datasets used and/or analysed during the current study are available from the corresponding author on reasonable request.

## Abbreviations

*B.pertussis*: *Bordetella pertussis*
Cp: Crossing points
Ct: Cycle threshold
hCPAP: Helmet continuous positive airway pressure
HFNC: High Flow Nasal Cannula
ICU: Intensive care unit
IQR: Interquartile range
LoD: Limit of detection
Tdap: Tetanus, Diphtheria, Pertussis Vaccine
