# Supplementary material for: A new epidemic wave of Bordetella pertussis in paediatric population: impact and role of co-infections in pertussis disease
Source: Ital J Pediatr. 2025 Jan 21;51:7. doi: 10.1186/s13052-025-01865-4 (PMC11749163; doi:10.1186/s13052-025-01865-4)
Supplement: Supplementary file 1 — Supplementary Material 1: Supplementary figure 1. Temporal distribution of B.pertussis in the pediatric population from January 01, 2024 to May 31, 2024 admitted to the Bambino Gesù Children's Hospital in Rome, Italy. [file 13052_2025_1865_MOESM1_ESM.pdf]

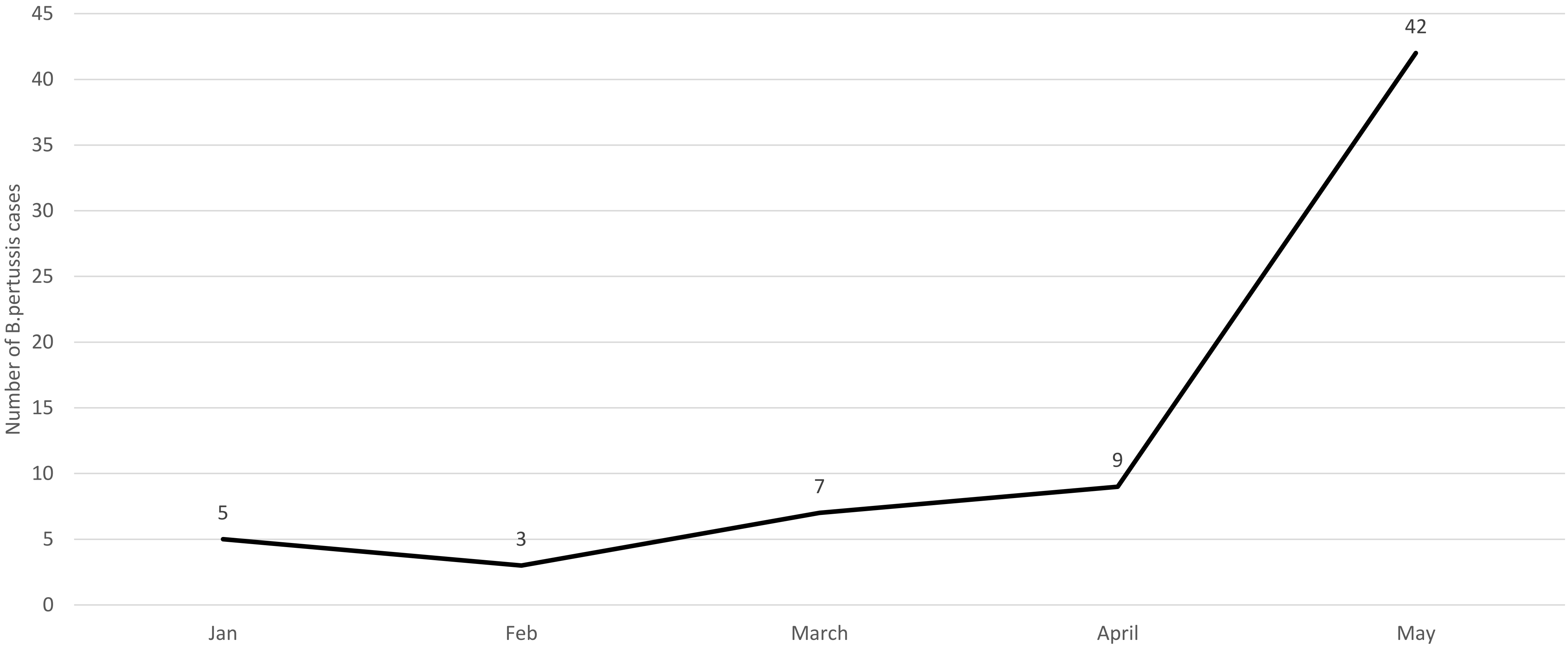

Supplementary figure 1. Temporal distribution of *B.pertussis* in the pediatric population from January 01, 2024 to May 31, 2024 admitted to the Bambino Gesù Children's Hospital in Rome, Italy.
